# Effects of acupuncture on the brain in primary insomnia: a coordinate-based meta-analysis of fMRI studies

**DOI:** 10.3389/fneur.2023.1180393

**Published:** 2023-07-18

**Authors:** Shuhan Zang, Ying Chen, Haonan Chen, Huawei Shi, Li Zhou

**Affiliations:** ^1^The First School of Clinical Medicine, Beijing University of Chinese Medicine, Beijing, China; ^2^Dongzhimen Hospital, Beijing University of Chinese Medicine, Beijing, China

**Keywords:** primary insomnia (PI), acupuncture, functional magnetic resonance imaging, amplitude of low-frequency fluctuations, regional homogeneity, systematic review

## Abstract

**Importance:**

Primary insomnia (PI) has a high global incidence, and effective treatments with fewer side effects are needed. Acupuncture, a treatment used in traditional Chinese medicine, has become increasingly established as a treatment method for PI and is recognized by many physicians and patients. Some evidence has suggested that acupuncture was associated with improvements in objective sleep parameters and might induce changes in some brain regions. Individual studies with limited sample size and low detection thresholds may lead to false positives, and no systematic review of the effects of acupuncture has been conducted in PI.

**Objective:**

The aim of this systematic review and coordinate-based meta-analysis was to summarize the literature on fMRI evaluation of patients with PI treated with acupuncture.

**Design:**

We performed a methodical and comprehensive search of multiple publication databases (from inception to December 2022): Web of Science, PubMed, ScienceDirect, Embase, Wan Fang, China National Knowledge Infrastructure, and Chinese Scientific Journal Database. Bias and quality of studies were evaluated by three researchers. Furthermore, a seed-based D-mapping meta-analysis with permutation of subject images (SDM-PSI) was applied to investigate the central mechanisms behind acupuncture treatment at PI. The International Prospective Registry of Systematic Reviews received the protocol for this study. (PROSPERO: CRD42023400086).

**Results:**

The analysis included 305 patients with PI and 116 healthy controls from 11 studies. SDM-PSI analysis showed that patients with PI exhibited increased amplitudes of regional homogeneity and low-frequency fluctuations in the left superior frontal gyrus (1352 voxels, *p* = 0.0028), right angular gyrus (14 voxels, *p* = 0.0457), and cerebellum (12 voxels, *p* = 0.0446). Acupuncture improved the function of right superior frontal gyrus (1, 404 voxels, *p* = 0.0123), left inferior frontal gyrus (1068 voxels, *p* = 0.0088), left inferior temporal gyrus (903 voxels, *p* = 0.0074), left supramarginal gyrus (888 voxels, *p* = 0.0113), left precuneus (457 voxels, *p* = 0.0247), right precuneus (302 voxels, *p* = 0.0191), left supplementary motor area (82 voxels, *p* = 0.0354), and right parahippocampal gyrus (28 voxels, *p* = 0.0379). The brain regions affected by non-acupoint acupuncture were all located in the frontal lobe. The Cochrane risk-of bias tool and MINORS5 were used for quality assessment and the included articles had high performance bias and attrition bias.

**Conclusion:**

This coordinate-based meta-analysis found that acupuncture in patients with PI had significant effects on the default mode network, particularly on the frontal lobe and precuneus, and that non-acupoint acupuncture may provide some benefit to frontal brain region function.

**Systematic review registration:**

PROSPERO: CRD42023400086.

## 1. Introduction

Primary insomnia (PI), typically characterized by problems of falling asleep and staying asleep, occurs in 6–10% of the global population (1). The persistent state of PI not only impairs daytime performance but also increases the risk of several psychiatric disorders, especially depression and anxiety (2). In recent years, several high-quality clinical trials have addressed the treatment of PI (3, 4).

Currently, medications and cognitive behavioral therapy are used to treat insomnia (5). Sleep medications may cause serious side effects, including dependence, cognitive deficits, confusion, and falls, so researchers are exploring other effective options with fewer side effects (6). Acupuncture, a traditional Chinese medicine procedure used in which sterile needles are inserted into specific areas of the body, is often used to treat PI because of its low side effects. This treatment method regulates mood and insomnia symptoms and is consistent with the contemporary biological–psychological– social–medical philosophy. Several systematic reviews have shown that acupuncture was associated with improvements in some objective sleep parameters (total sleep time, sleep efficiency, and number of awakenings) (7), and that treatment of more than 3 weeks could significantly improve symptoms (8). However, the certainty of the evidence was moderate to low.

Recently, with the popularization of neuroimaging methods, more researchers have focused on structural and functional changes in the brains of PI patients. Functional magnetic resonance imaging (fMRI), a technique that assesses hemodynamics using blood oxygen level-dependent signals, is commonly used to study changes in the brain. Assessment of spontaneous neuronal activity in the blood oxygenation level-dependent (BOLD) signal can be achieved by calculating regional intensity at rest using measures such as amplitude of low-frequency fluctuation (ALFF) and regional homogeneity (ReHo) (9, 10). Central nervous system changes in patients with PI tend to focus on brain regions associated with emotional and cognitive functions, and these changes have been linked to the onset, development, and severity of the disorder. For instance, insomnia symptoms in patients with PI may be associated with decreased ReHo levels in the amygdala, cingulate cortex, and frontal lobe, and decreased ALFF levels in the thalamus. These changes may correspond to high levels of introspection, worry, and rumination (11). Several studies have demonstrated that appropriate treatment of PI can reverse structural and functional brain abnormalities in patients. The function of several brain regions was shown to change in patients with PI after cognitive behavioral therapy (12), treatment with benzodiazepine, and cupping (13).

Previous studies on the mechanisms of acupuncture have shown that acupuncture can affect brain activity and have long-lasting and significant effects on neural functions (14). As the number of clinical trials using acupuncture to treat PI continues to increase, a number of studies have confirmed the hypothesis that the activity of specific brain areas was related to sleep and shown that acupuncture could alter these regions (15, 16). However, single studies with small samples and low detection thresholds may lead to false positive results, and there are no systematic reviews of how acupuncture or non-pharmacological treatments affect brain imaging characteristics in PI patients.

The aim of this systematic review and coordinate-based meta-analysis was to summarize the current understanding of regional brain fMRI imaging features in patients with PI treated with acupuncture. To this end, we summarized the coordinates of ReHo and ALFF in qualified studies and used Seed- based D Mapping with Permutation of Subject Images (SDM-PSI) for data processing, summarization, and analysis to systematically summarize the brain imaging features of patients with PI and evaluate the therapeutic effects of acupuncture.

## 2. Materials and methods

The Preferred Reporting Items for Systematic Reviews and Meta-Analyses guidelines (17) were observed by the conduct of the present study. The International Prospective Registry of Systematic Reviews received the protocol for this study. (PROSPERO: CRD42023400086).

### 2.1. Literature search

We performed a methodical and comprehensive search of the literature published between the establishment of the following publication databases until December 2022: Web of Science, PubMed, ScienceDirect, Embase, Wan Fang (WF), China National Knowledge Infrastructure (CNKI), and Chinese Scientific Journal Database (VIP). The following search terms were used under the “subject words plus free words” retrieval strategy: (“Acupuncture” OR “Needle” OR “Acupoint”) AND (“fMRI” OR “Magnetic resonance imaging” OR “FC” OR “ROI” OR “ReHo” OR “ALFF” OR “Neuroimaging” OR “Brain”) AND (“Insomnia” OR “Sleep Disorder”). In addition, all reference lists for each article, and all relevant review articles, were manually reviewed to identify additional potentially qualifying studies. The search process was performed individually by two authors (ZSH and CY).

#### 2.1.1. Eligibility criteria

The following inclusion criteria were used to screen all original studies published in English or Chinese:

Types of trials: Randomized controlled trials, non-randomized controlled trials, and observational studies;

Participants: Adult patients who met the diagnostic criterion for primary insomnia from the Diagnostic and Statistical Manual of Mental Disorders, 5th Edition (DSM-5);

Intervention: Various forms of acupuncture (characterized by the insertion of a needle into the skin surface of the acupoint, such as manual acupuncture, electroacupuncture, auricular acupuncture and needle warming moxibustion) or acupuncture in combination with other medications;

Outcomes: (1) Reporting an analysis of relevant data (patient data before and after acupuncture, comparisons with healthy subjects or non-acupoint acupuncture groups); (2) fMRI and subjective scales were used as parameters, with whole-brain imaging with function (ALFFs or ReHo) at rest serving as the primary outcome measure; (3) Whole-brain imaging results were displayed in stereotactic coordinates (Talairach or Montreal Neurological Institute [MNI]) in three dimensions (x, y, and z).

The following exclusion criteria were also used: (1) Participants included patients with insomnia comorbid with other conditions; (2) Animal experiments and duplicate work; (3) No available stereotactic peak coordinates; (4) Insufficient data for meta-analysis in the original study or after contacting the authors.

#### 2.1.2. Study selection

Duplicate studies were eliminated using NoteExpress software (v3.2.0.7276; Aegean Software Corp., Beijing, China). Studies that did not meet the inclusion criteria were then excluded based on the titles and abstracts of the remaining articles. In a final step, a thorough full-text review of potentiallyrelevant studies were performed. Two authors (ZSH and CY) assessed the articles separately, resolving any disagreements by consensus or consultation with a third principal investigator (CHN).

### 2.2. Data extraction

For each study, a graph was created with information on the following: (1) publication details (author, journal, year); (2) basic study details (type of study, comparisons, sample size, scanning instrument, functional data analysis); (3) acupuncture methods (acupuncture type, acupoints, duration of treatment, frequency, duration); (4) clinical outcome measures (Pittsburgh Sleep Quality Index scores before and after treatment); (5) neuroimaging results (peak coordinates [MNI or Talairach] and corresponding statistics (t scores, Z scores). If certain data were missing from the original manuscripts, the corresponding authors were contacted by e-mail for further information. Data entry and extraction were performed by two independent authors (ZSH and CY).

### 2.3. Quality assessment

We used risk-of-bias tools developed by the Cochrane Collaboration (18) (https://training.cochrane.org/handbook) to assess the quality of the literature, considering seven factors: creation of random sequences, allocation concealment, blinding of researchers and study participants, blinded assessment of study results, completeness of data, incomplete reporting, and other bias risk. MINORS5 (19) was used to assess the quality of non-randomized controlled trials. Quality assessment was carried out independently by two authors (ZSH and CY). In case of disagreement, a third author (CHN) was consulted.

### 2.4. SDM-PSI meta-analysis

Imaging data from the eligible articles (ReHo and ALFF) were analyzed using an SDM software package (SDM v6.22; www.sdmproject.com). Differences in brain activity between patients with PI and healthy subjects, before and after acupuncture treatment in patients with PI, and between real and sham acupuncture therapy was compared. Software instructions were strictly followed, and accurate and comprehensive data were expected (20). (1) A text file containing peak positions and effect dimensions (t value or z-score or *P*-value) was created by extracting the peak data. Talairach coordinates were also converted to MNI coordinates. (2) A new statistical chart was recreated separately for each data set in the standard MNI chart. (3) Mean maps were constructed from the means of the random effects datasets based on sample size, study variance, and between-group heterogeneity. (4) A quantitative meta-analysis was performed to compare fMRI changes between patients with PI and healthy subjects, and between patients with PI in the true acupuncture group before and after treatment. (5) The following default SDM parameters were used: anisotropy = 1, isotropic full width at half maximum = 20, peak Z = 1, and voxel size = 2. (6) MRIcron software was used to display all statistically significant results.

## 3. Results

### 3.1. Characteristics of included studies

**Figure 1:**
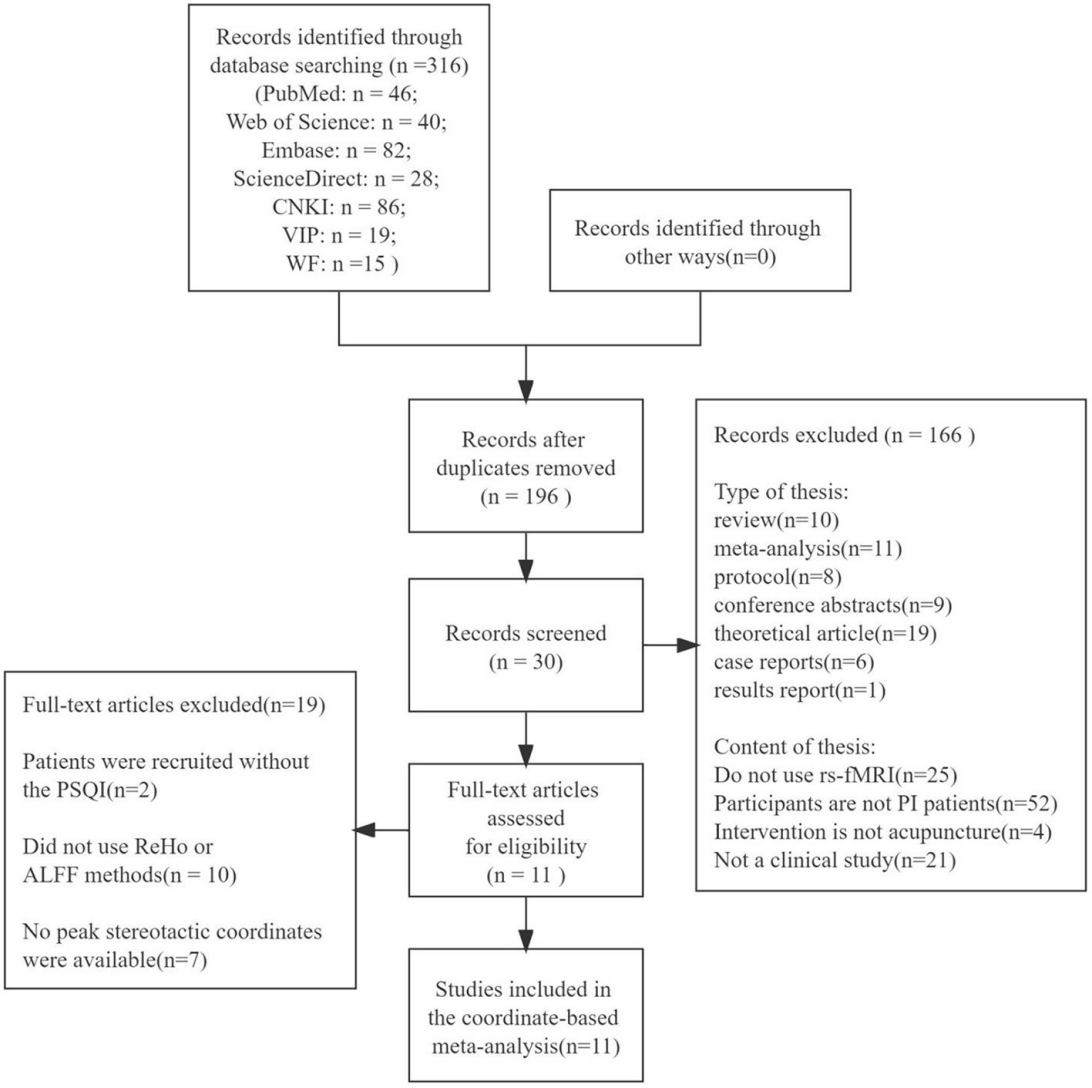
Flow diagram of literature search.

An initial search identified 316 articles, comprising 196 articles in English and 120 in Chinese. After screening the type and content of these articles, 11 studies (9 in Chinese and 2 in English) were finally included. An illustration of the article selection process can be found in Figure 1.

The included studies were published from 2016 to 2021. A total of 116 healthy controls were compared to 305 patients with PI. Table 1 represents essential details of the eligible articles, summarized as follows. (1) Study types: the selected studies included five randomized controlled trials, three unequal group-controlled trials (healthy subjects formed control groups), and three self-controlled trials. (2) Participants: all studies recruited patients diagnosed as PI according to the DSM-5 criteria; three of them recruited healthy control subjects, and demographic data of all included healthy controls were matched with those of patients in each study. (3) Intervention: all interventions in the treatment groups were acupuncture, including manual acupuncture, electroacupuncture, and auricular acupuncture; five studies used non-acupoint acupuncture as control treatment. (4) Age and gender: most of the subjects included in the studies were middle-age and elderly, with significantly more women than men. (5) Acupuncture methods and acupoints: Pure acupuncture was used in five studies, auricular acupuncture in three, electroacupuncture in two, and both acupuncture and fire acupuncture in one; the most frequently chosen acupoint was Shenmen (HT-7), followed by Baihui (GV-20) and Sanyinjiao (SP-6). In addition, acupoints regulating liver and spleen were also used. (6) Outcome measures: ReHo and ALFF were measured by fMRI in all studies; nine studies presented data on alterations in brain regions in PI patients both before and after an acupuncture session, three articles presented comparisons of brain regions between patients with PI and healthy controls, and we also found imaging findings from non-acupoint acupuncture in patients with PI and/or comparisons of brain area changes in four studies.

**Table 1:** Characteristics of the included studies.

**References**	**Type of research**	**Sample size**	**Type of acupuncture**	**Age (Years); Gender (F:M)**	**Main acupoints**	**Frequency of acupuncture**	**Days of treatment**	**Analysis method**
Shi et al. (16)	ST	VA (30)	EA	Gender: F: 18; Age: 52.88 ± 4.55; M: 12; Age: 45.00 ± 12.70	HT-7	QD	35	fALFF
		VA1 (5)		Age: 51.45 ± 12.10; Gender: 4:1	HT-7, SP-6, GV-20	QD	35	fALFF
Wang et al. (15)	RCT	PCs: VA2 (5)	EA	Age: 55.42 ± 9.23; Gender: 3:2	HT-7	QD	35	fALFF
		PCs: NAA (5)		Age: 51.10 ± 11.60; Gender: 4:1	Non-acupoint	QD	35	fALFF
Luo (21)	UCT	VA (72)	AA	Age: 48 (35~57); Gender: 51:21	CO12, CO15	BID	28	fALFF, ReHo
		HCs: 74		Age: 46.5 (33~52); Gender: 57:17				
He et al. (22)	ST	VA (20)	AA	Age: 42.5 ± 15.4; Gender: 12:8	CO10, CO15	QD	28	ALFF, fALFF, R
		VA (15)		Age: 52.13 ± 14.05; Gender: 9:6	HT-7, KI-7			
Wang (23)	RCT	PCs: NAA (15)	MA	Age: 50.73 ± 14.88; Gender: 11:4	QOD	20	ALFF, fALFF	
		VA1 (10)		Age: 51.45 ± 12.10; Gender: 8:2				
Wang (24)	RCT	PCs: VA2 (10)	EA	Age: 55.42 ± 9.23; Gender: 7:3	HT-7	QD	35	ALFF
		PCs: NAA (10)		Age: 51.10 ± 11.60; Gender: 8:2	Non-acupoint			
		VA1 (9)	MA, FA	Age: 52.56 ± 12.23; Gender: 7:2	HN3, Sishencong, Anmian, BL-62, KI-6, BL-15, BL- 20, RN12, ST-36	QD	14	ALFF, ReHo
Ding (25)	ST	PCs: VA2 (8)	MA	Age: 49.38 ± 10.43; Gender: 6:2	HT-7, EX- HN3, Sishencong, Anmian, BL-62, KI-6, BL-15, BL-20			
		VA (16)		Age: 33.2 ± 4.94; Gender: 11:5	HT-7, SP-6, GV-20,KI-6,BL-62, Anmian,BL-15,BL-20,ST-36			
Zhang (26)	RCT	PCs: NAA (12)	MA	Age: 31.8 ± 4.91; Gender: 10:2	Non-acupoint			
Huang (27)	UCT	VA (32)	MA	Age: 41.38 ± 8.32; Gender: 20:12	GB-20, ST-40, LR-3	QD	30	HCs: 20
		HCs: 20		Age: 41.12 ± 8.20; Gender: 12:8				
Huang and Wang (28)	RCT	VA (6)	MA	Age: 41.13 ± 10.51; Gender: 4:2	BL-18, BL-20, LR-3, SP-3, LR- 2, SP-2	TIW	21	ALFF, fALFF, R
		PCs: NAA (6)		Age: 39.89 ± 9.06; Gender: 3:3	Non-acupoint			
Xu et al. (29)	UCT	VA (29)	MA	Age: 42.1 ± 8.4 Gender: 19:10	GV-20, Sishencong, HT-7, SP-6	QD	14	
		HCs: 22		Age: 38 ± 10.5 Gender: 16:6				ReHo

VA, verum acupuncture; NAA, Non-acupoint acupuncture; PCs, patient control; HCs, health controls; MA, manual acupuncture; EA, electroacupuncture; FA, fire acupuncture; AA, auricular acupuncture; ST, Self-control test; UCT, Unequal group-controlled trial; RCT, Randomized controlled trial; ReHo, regional homogeneity; ALFF, amplitude of low-frequency fluctuations; fALFF, fractional amplitude of low-frequency fluctuations; QD, once a day; BID, twice a day; QOD, once every other day; TIW, three times a week.

### 3.2. Quality assessment

**Figure 2:**
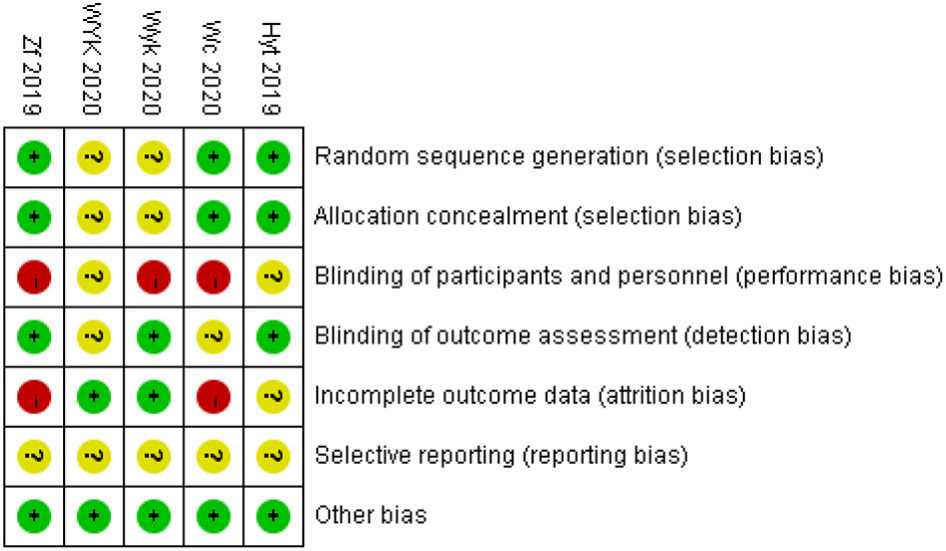
Quality assessment of randomized controlled trials.

The Cochrane risk-of bias tool was used for quality assessment, and there were seven entries in the evaluation criteria. In all included randomized controlled trials, allocation concealment and blinding were not discussed, resulting in a high level of performance bias. In five randomized controlled trials, three studies discussed the process of generating random sequences. Some missing data resulted in high attrition bias. We did not detect any other factors leading to bias. Figure 2 illustrates this assessment. Quality assessment of non-randomized controlled trials was performed using MINORS5, which is also shown in Table 2.

**Table 2:** Quality assessment of non-randomized controlled trials.

**Number**	**Entries**	**Study**	**Shi et al. (16)**	**Luo et al. (21)**	**He et al. (22)**	**Ding (25)**	**Huang (27)**	**Xu et al. (29)**
1	A clearly stated aim		2	2	2	2	2	2
2	Inclusion of consecutive patients		1	1	1	1	1	0
3	Prospective collections of data		1	1	0	0	0	0
4	Endpoints appropriate to the aim of the study		2	2	2	2	2	2
5	Unbiased assessments of the study endpoint		0	1	1	2	1	1
6	Follow-up period appropriate to the aim of the study		2	2	2	2	2	2
7	Loss to follow up < 5%		2	0	0	0	2	1
8	Prospective calculations of the study size		0	2	0	2	0	0
9	aggregate score		10	11	8	11	10	8
10	Additional criteria in the case of comparative studies							
11	An adequate control group			2		2	2	2
12	Contemporary groups			2		2	2	2
13	Baseline equivalence of groups			1		2	0	1
14	Adequate statistical analyses			2		2	2	2
15	aggregate score			18		19	16	15

The items are scored 0 (not reported), 1 (reported but inadequate), or 2 (reported and adequate), with the global ideal score being 16 for non-comparative studies and 24 for comparative studies.

### 3.3. Results of meta-analysis

#### 3.3.1. Comparison of brain regions between patients with PI and healthy controls

We found a significant increase in activity in the left superior frontal gyrus in patients with PI compared to healthy subjects (1352 voxels, *p* = 0.0028). Signals in the right angular gyrus (14 voxels, *p* = 0.0457) and cerebellum (12 voxels, *p* = 0.0446) were also elevated.

#### 3.3.2. Acupuncture manipulation of brain areas in patients with PI

According to our analysis, acupuncture resulted in increased signals in some brain regions, with the largest cluster (1, 404 voxels, *p* = 0.0123) involving the right superior frontal gyrus, followed by the left inferior frontal gyrus (1, 068 voxels, *p* = 0.0088). Other brain regions affected included left inferior temporal gyrus (903 voxels, *p* = 0.0074), left supramarginal gyrus (888 voxels, *p* = 0.0113), left precuneus (457 voxels, *p* = 0.0247), right precuneus (302 voxels, *p* = 0.0191), left supplementary motor area (82 voxels, *p* = 0.0354), and right parahippocampal gyrus (28 voxels, *p* = 0.0379); all showed an increase in activity after acupuncture.

#### 3.3.3. Non-acupoint acupuncture manipulation of brain areas in patients with PI

Non-acupoint acupuncture had an effect in some brain regions, involving few voxels that increased their signal. These brain regions were concentrated in the frontal lobe, including the left superior frontal gyrus (298 voxels, *p* = 0.0044), left inferior frontal gyrus (227 voxels, *p* = 0.0088), and right inferior frontal gyrus (122 voxels, *p* = 0.0404).

#### 3.3.4. Differences in effects between acupuncture and non-acupoint acupuncture

**Figure 3:**
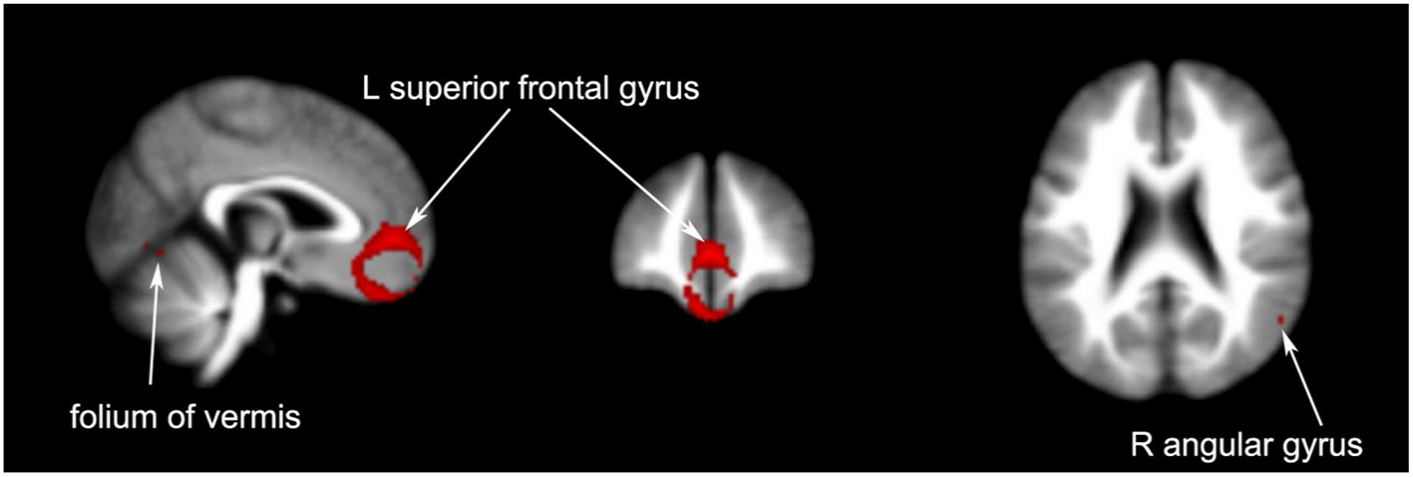
Brain regions with increased signals in patients with PI compared to healthy controls.

**Figure 4:**
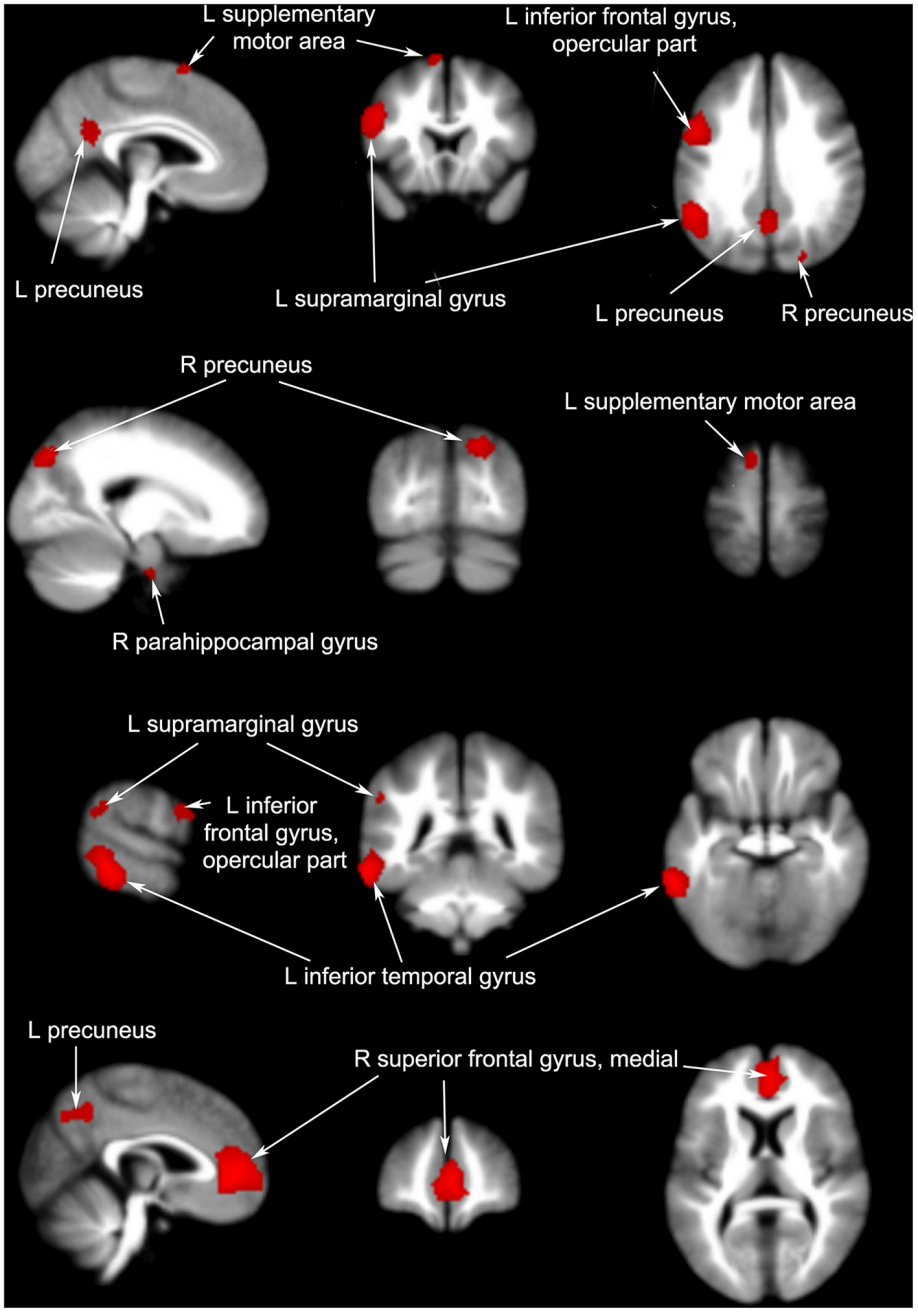
Brain regions with increased signals pre- and post-acupuncture in patients with PI.

**Figure 5:**
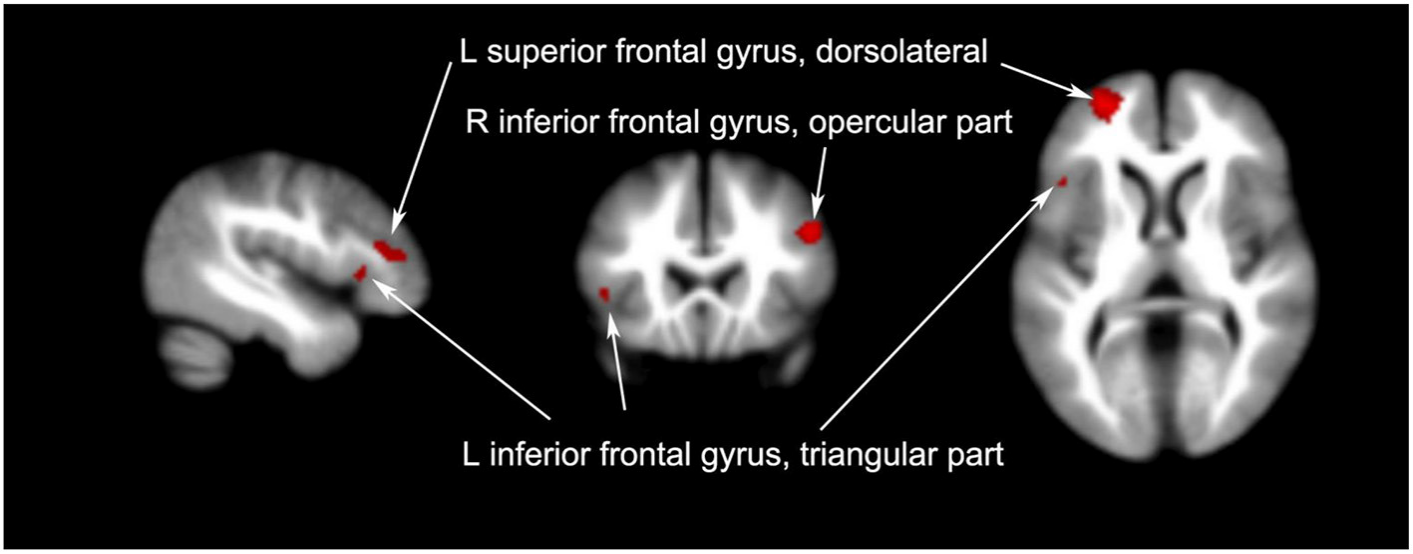
Brain regions with increased signals before and after non-acupoint acupuncture in patients with PI.

**Figure 6:**
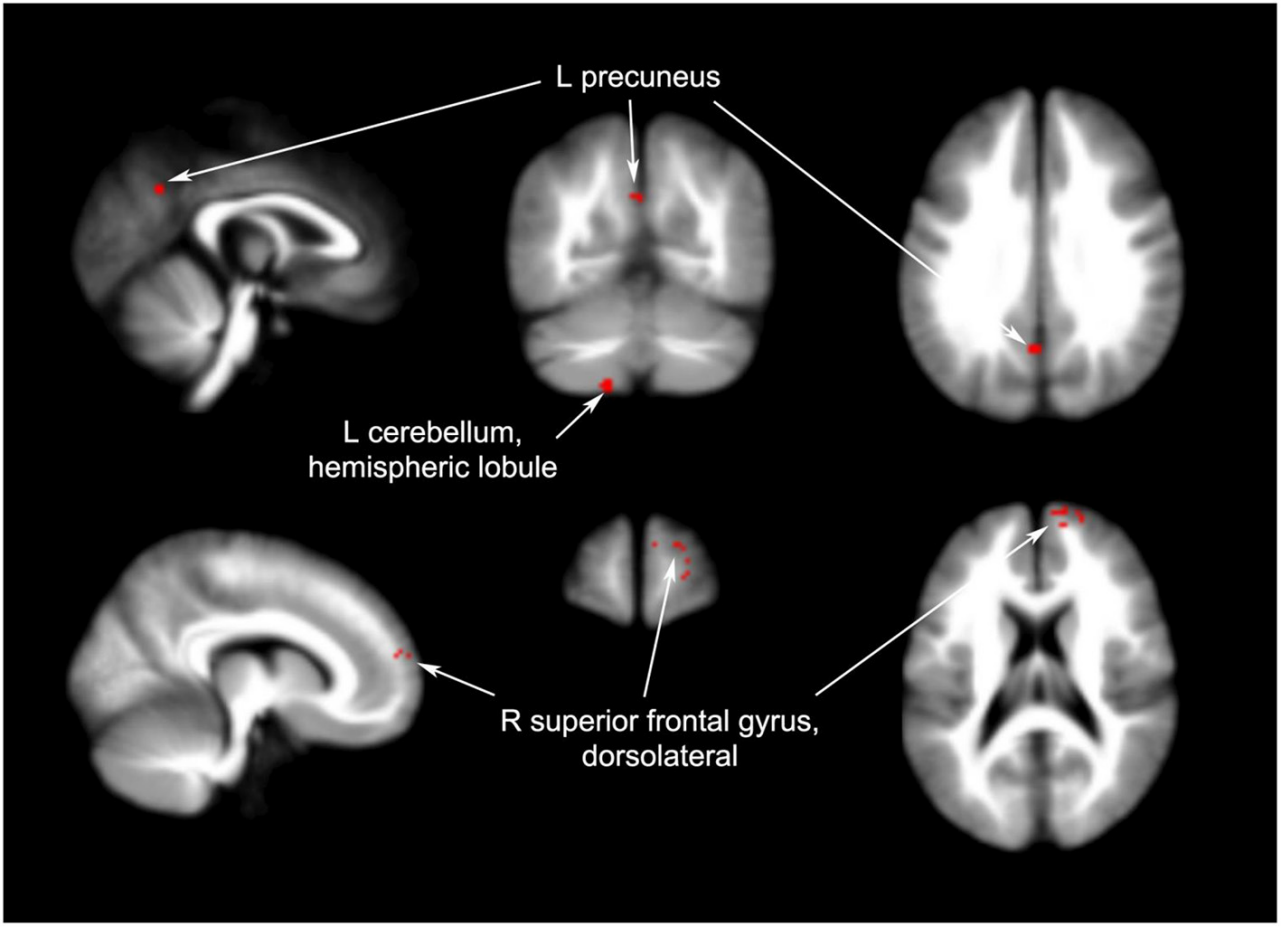
Brain regions with increased signals in acupuncture groups compared to non-acupuncture groups.

Four articles compared different brain regions affected by acupuncture and non-acupoint acupuncture including the left cerebellum (40 voxels, *p* = 0.0491), right superior frontal gyrus (22 voxels, *p* = 0.0454), and left precuneus (12 voxels, *p* = 0.0456). The results of the coordinate meta-analysis are shown in Table 3 and Figures 3–6 in detail.

**Table 3:** Results of the coordinate meta-analysis.

**Comparison**	**Brain Regions**	**MNI**			**SDM–Z**	**P**	**Voxels**	**Sensitivity analysis**
		**x**	**y**	**z**				
Patients vs. Healthy	Left superior frontal gyrus, orbital part, BA 11	−8	44	−24	2.771	0.0028	1352	2/3
	Right angular gyrus, BA 39	50	−62	26	1.688	0.0457	14	1/3
	Cerebellum, vermic lobule VI	6	−66	−8	1.700	0.0446	12	1/3
Before and after acupuncture in patients with PI	Left inferior frontal gyrus, opercular part, BA 44	−50	8	28	2.373	0.0088	1068	4/9
	Right superior frontal gyrus, medial, BA 10	4	46	0	2.246	0.0123	1, 404	6/9
	Left inferior temporal gyrus, BA 20	−62	−42	−16	2.435	0.0074	903	1/9
	Left supramarginal gyrus, BA 40	−52	−50	34	2.279	0.0113	888	5/9
	Left precuneus	0	−56	34	1.965	0.0247	457	5/9
	Right precuneus, BA 19	18	−76	42	2.071	0.0191	302	4/9
	Left supplementary motor area, BA 6	-8	12	66	1.807	0.0354	83	4/9
	(undefined), BA 36	22	−8	−36	1.775	0.0379	28	3/9
Before and after 45 non–acupoint acupuncture in patients with PI	Left superior frontal gyrus, dorsolateral, BA 10	−24	56	8	2.623	0.0044	298	3/3
	Left inferior frontal gyrus, triangular part, BA	−50	34	14	2.374	0.0088	227	2/3
	Right inferior frontal gyrus, opercular part, BA 44	48	20	30	2.345	0.0095	122	2/3
	Left inferior frontal gyrus, triangular part, BA 47	−44	22	2	1.746	0.0404	10	2/3
Acupuncture group vs. non-acupuncture group	Left cerebellum, hemispheric lobule VIII	−16	−58	−58	1.654	0.0491	40	2/4
	Right superior frontal gyrus, dorsolateral, BA 10	22	62	16	1.691	0.0454	22	1/4
	Left precuneus	0	−58	30	1.689	0.0456	12	2/4

## 4. Discussion

### 4.1. Overview of the results

Focusing on changes in ReHo and ALFF, we summarized the current state of research and the effects of acupuncture on brain areas in PI patients based on neuroimaging data. In total, 11 fMRI studies on coordinates were pooled using the SDM-PSI meta-analysis method, and we uncovered some meaningful results. One notable finding was that frontal lobe activation appears to be both a key pathological feature of patients with PI and an important mechanism for the effect of acupuncture on insomnia. In addition, the precuneus, supramarginal gyrus, and supplementary motor area in patients with PI showed increased resting-state activity after acupuncture treatment. We also found that most of the brain areas affected by acupuncture were primarily associated with the default mode network (DMN).

One of the accepted theories is that PI is accompanied by some form of unique hyperarousal (30). In accordance with this, fMRI results have indicated increased activity and disinhibition, as well as increased instability of intrinsic activity in PI (31). Summarizing the three studies included here with healthy controls (133 patients with PI vs. 116 healthy controls), patients with PI had higher ALFF and ReHo levels in the left superior frontal gyrus, right angular gyrus, and cerebellum. No brain areas with low ReHo and ALFF levels were found, which was highly in keeping with this theory. This did not seem to be entirely consistent with previous studies (32), possibly due to the small sample size. Unexpectedly, several brain regions with increased ALFF and ReHo levels were also found after both acupuncture and non-acupoint acupuncture treatment, which were associated with a reduction in insomnia symptoms. In conjunction with the associations between hyperarousal in insomnia and neurocognitive deficits (33), we hypothesized that there might be a “spurious active” state in certain brain regions in patients with PI and that acupuncture treatment could actually improve the function of these regions.

### 4.2. Effects of acupuncture on the frontal lobe of patients with PI

The main function of the frontal lobe is to integrate signals from other brain regions, and this area is highly involved in understanding of past and current experiences and developing plans for future actions (34). Previous studies have shown that maturation of the frontal lobe was related to sleep status (35). The present results showed that significant changes in the frontal lobe occurred not only in patients with PI compared to healthy controls, but also when comparing patients with PI before and after treatment, indicating the important role of frontal lobe modifications in the neuropathological changes associated with PI.

The prefrontal lobe is not only involved in linking external and internal environments and identifying episodic memories, but also works with other parts of the brain to maintain resting state network activity (36). Studies have shown that difficulty falling asleep may be related to a lack of reduction in activity in the prefrontal cortex (37) and that people with insomnia showed a slower decrease in relative cerebral metabolic rates of glucose in the medial prefrontal cortex during the transition from wakefulness to non-REM sleep compared to controls (38). The orbitofrontal lobe, located in the prefrontal lobe, may influence the regulation of emotional memories during sleep (39). In the present statistical results, the orbitofrontal lobe showed the most significant difference when comparing patients with PI and healthy individuals, suggesting that this may be a crucial brain region associated with insomnia symptoms.

Two parts of the frontal lobe, the left inferior frontal gyrus (opercular part) and the right superior frontal gyrus (medial), changed significantly before and after acupuncture. The left inferior frontal gyrus has a crucial function in phonological processing and verbal working memory (40). The right superior frontal gyrus has a specific role in translating conflict anticipation into the control of impulsive responses, and its activation is associated with more effective response inhibition and a lower sense of motor urgency (41). It is possible that changes in these two brain regions are responsible for the improved information processing ability after acupuncture in patients with PI.

The frontal lobe was the most frequently changed brain region in patients with PI following non- acupoint acupuncture, but the number of changed voxels in all brain regions was much lower than after acupuncture treatment. The changes occurred in the left superior frontal gyrus (dorsolateral), left inferior frontal gyrus (triangular part), and right inferior frontal gyrus (opercular part). The dorsolateral circuit is thought to control attention, planning, and organization (33). The triangular part of the inferior frontal gyrus serves as a connection point between the DMN, the sensory/somatomotor network, and the dorsal and ventral attention networks (42). The placebo effect of non-acupoint acupuncture could promote the perception of improved sleep in patients with PI and increase their confidence in their sleep quality.

In general, both acupuncture and non-acupoint acupuncture enhanced frontal brain activity in patients with PI. Frontal lobe function is involved in a number of cognitive processes, including problem solving, working memory, planning, and evaluation of goal-oriented activities. Enhancing its function may help alleviate the subjective insomnia and environmental discomfort experienced by people with PI, thereby reducing their pre-sleep worries and rumination (37). Compared with the results of non-acupoint acupuncture, it can be concluded that acupoint acupuncture had a real effect on the function of the frontal lobe function.

### 4.3. Effects of acupuncture on the default mode network in patients with PI

The default mode network (DMN) is activated when people are left alone to contemplate and comprises a group of interconnected brain regions involved in cognition (43). The prefrontal cortex, precuneus, angular gyrus, posterior cingulate cortex, and transverse parietal cortex make up the most of the DMN (44). During deep sleep, certain DMN regions dissociate, suggesting that connectivity in this network may support certain states of consciousness (45). Based on the understanding of self-referential stress in patients with PI, one theory is that insomnia may be caused by the disruption or imbalance of DMN activity, and previous studies have confirmed that it appears to be altered or imbalanced in insomnia (46).

In terms of function, the DMN can be divided into two parts: the pre-DMN, which is centered on the prefrontal lobe, and the rear-DMN, which is centered on the precuneus and angular gyrus (47). The pre-DMN is involved in psychological behaviors that are self-referential, while the post-DMN is involved in memory of past experiences (48). The effects of acupuncture on the prefrontal lobe were discussed above and suggested that the function of the pre-DMN was altered by acupuncture. The precuneus, the functional core of the rear-DMN, is a brain area that helps remember events from the past, which is fundamental to our self-esteem and interactions with others (49). The function of the precuneus is bidirectional in relation to sleep status. People with increased blood oxygen level-dependent signals in the bilateral precuneus showed high subjective–objective discrepancy of sleep (50), and the functional connectivity density of the precuneus decreased after sleep deprivation (51). The present study showed that acupuncture affected the function of the left and right precuneus in patients with PI, and the precuneus also appeared in comparisons between acupuncture and non-acupoint acupuncture, suggesting that acupuncture can indeed alter its function. With the exception of the prefrontal lobe and the precuneus as mentioned above, other brain areas affected after acupuncture were the supramarginal gyrus and inferior temporal gyrus, which are also part of the DMN. This seemes to indicate that acupuncture improved the function of the DMN in patients with PI.

Impairments associated with insomnia can be classified into cognitive, self-referential, affective, and sleep–wake promotion domains (52), among which DMN dysfunction primarily affecting cognition and self-referential systems. The present results suggest that acupuncture could act on the DMN to improve self-awareness and perception of the environment and reduce the occurrence of their bad memories in patients with PI, affecting their adaptability to the environment and their perception of sleep.

### 4.4. Strengths and limitations

To our knowledge, the present study was the first to review the results of fMRI brain imaging associated with acupuncture in patients with PI using a coordinate-based meta-analysis. Here, we presented new evidence of neuroplasticity induced by acupuncture in a neurological disorder. Precise inclusion criteria were established to avoid selection bias, and 11 studies were thoroughly read and filtered before being pooled in the final analysis. Ultimately, we identified some meaningful results that provide a foundation and challenges for future scientific research.

However, this meta-analysis has certain limitations. First, we included only 11 studies and did not test for heterogeneity. Because of the limited sample size of the listed studies, there were large differences among the studies in the design of the clinical study protocol, the choice of acupoints, and the methods of analysis, and some results were not reproducible, which significantly affected the reliability of the results. As a result, caution should be exercised in interpreting the results. In most studies, the methods of blinding and concealment of group assignment were not accurately described, therefore the results had some degree of methodological bias. Finally, only publications in Chinese and English languages were used, which may have influenced the results and affected the usefulness and reliability of the present study.

Larger sample numbers and more rigorous randomized controlled trials are needed in future studies to improve reproducibility and accuracy. To fully explore the true therapeutic effect of acupuncture, future clinical trials should standardize sham acupuncture groups and apply more uniform implementation standards for device selection, patient characteristics at baseline, and outcome assessment.

## 5. Conclusions

The present coordinate-based meta-analysis found that the impact of acupuncture in patients with PI was mainly concentrated in the DMN, especially in the frontal lobe and precuneus, and non-acupoint acupuncture also showed some benefits in the frontal brain. This work provided a new perspective to clarify the function of brain areas in acupuncture treatment of PI. To ensure the reliability of future clinical data, large-scale and rigorous randomized controlled trials should be conducted.

## Data availability statement

The original contributions presented in the study are included in the article/supplementary material, further inquiries can be directed to the corresponding author.

## Author contributions

SZ, YC, HC, and LZ: concept and design. SZ, YC, and HC: acquisition of data. SZ and YC: drafting of the manuscript. LZ and HS: critical revision of the manuscript. All authors agree to be accountable for all aspects of the work, contributed to the article and approved the submitted version.
